# Waterbird guilds function as dynamic cross-ecosystem energy vectors in unique soda pan model systems

**DOI:** 10.1038/s41598-026-57369-6

**Published:** 2026-06-20

**Authors:** Emil Boros

**Affiliations:** Department of Geography and Natural Sciences, Ludovika University of Public Service, Ludovika sqr. 2, 1083 Budapest, Hungary

**Keywords:** Avian energy transport, Basal metabolic rate, Field metabolic rate, Net energy import, Net energy export, Percent energy balance, Ecology, Ecology, Environmental sciences

## Abstract

Migratory animals play a key role in coupling ecosystems through the redistribution of energy, to date, energy transport by functional guilds has not been comprehensively quantified. Here, I present a generalized, own guild-based framework that extends established waterbird nutrient-cycling guild concepts into the energy flow across ecosystems, enabling the quantification of energy import, export and net balance mediated by waterbirds. Using long-term (1986–2017) waterbird census data from two internationally important (Ramsar-sites) intermittent soda pans in Central Europe, as simplified unique model systems. I estimated avian energy fluxes by integrating guild-specific foraging behaviour, habitat use, metabolic demand and residence time. Waterbird guilds were classified as net importers, importer–exporters or net exporters of energy based on Boros’s method. Estimated annual energy fluxes varied across several orders of magnitude, reflecting pronounced interannual variability in the studied ecosystem. Net energy import ranged widely from 574,712 to 57,022,011 kJ/ha/yr, while net energy export from 361,206 to 16,171,538 kJ/ha/yr. The net energy import consistently and significantly exceeded net energy export. Consequently, the average community-level energy balance was significantly positive (+ 51.3) regarding waterbird-mediated nutrient transport. The functional asymmetry between guilds ensures that energy import remains dominant over export regardless of species richness, fundamentally defining the ecosystem’s role as a significant energy sink in the studied system. Temporal analyses revealed significant dynamics in net energy import, whereas variation in open water area did not predict exactly energy balance. I partly reject the hypothesis that net energy import responds predictably to long-term trends in open water area, but partly confirm as it is rather depends on guild-structure dynamics. These results demonstrate that cross-ecosystem energy transport by waterbirds is governed primarily by functional guild composition and behaviour rather than habitat extent alone. The proposed framework provides a scalable tool for integrating avian-mediated energy transport into ecosystem energetics and spatial food-web theory.

**Supplementary Information:**

The online version contains supplementary material available at https://doi.org/10.1038/s41598-026-57369-6.

## Introduction

Migratory animals are increasingly recognised as powerful agents of cross-ecosystem connectivity, redistributing nutrients, organic matter and energy as they move between habitats^1, 2^. Among vertebrates, waterbirds function as highly mobile trophic integrators, transfer energy and nutrients across aquatic–terrestrial interfaces through predictable commuting between spatially distinct feeding, roosting and breeding sites, often over migratory timescales. Through these movements, waterbirds can stimulate primary production, restructure food webs and modify biogeochemical processes at local and landscape scales, and thereby exert a significant influence on the magnitude, direction and temporal dynamics of energy flow between ecosystems^3, 4, 5, 6, 7, 8^. Despite this well-established importance for spatial ecology, waterbirds are still most often conceptualised as consumers within local food webs, and a general framework capable of predicting how entire waterbird assemblages function as directional vectors of cross-ecosystem energy transport remains lacking.

Existing studies have focused largely on nutrient redistribution, guanotrophication or species-specific bioenergetics. Research on nutrient vectors has quantified allochthonous nitrogen and phosphorus loading by external feeders^9, 10, 11, 12, 13^, while studies of seabird and waterbird colonies have demonstrated pronounced long-term ecosystem modification through guano deposition^14, 15, 16^. Bioenergetic studies have evaluated energetic intake and expenditure for individual species^17, 18^, yet these approaches do not scale to communities and therefore cannot predict how variation in functional composition shapes ecosystem-scale energy balance. Recent advances in nutrient-cycling guild classification demonstrate that waterbirds can be grouped according to their theoretical net contribution to nutrient import, export or internal recycling^19, 20, 21^, providing a behavioural basis for community-level inference. However, these models remain restricted to mass fluxes (C, N, P) and do not quantify how guild-specific movements reshape energy flow, as fundamental metric of trophic dynamics.

Energy flux is central to ecological theory, underpinning food-web stability, trophic efficiency and ecosystem functioning^22, 23^. Yet no general framework exists for predicting cross-ecosystem energy import and export driven by waterbirds or other migratory vertebrate guilds. Specifically, a model is lacking that integrates (i) guild-specific behavioural time budgets, (ii) spatial feeding–roosting distributions, (iii) terrestrial–aquatic energy transport, and (iv) both energy import, export and net energetic balance at the ecosystem scale. This represents a critical conceptual and methodological gap in understanding how animal movement couples ecosystems and regulates emergent trophic patterns^24^.

The main goal of this study was to quantify the magnitude and temporal variability of waterbird-mediated energy import, export and balance in two soda pans over a multi-decadal period using a waterbird nutrient-cycling guild framework extended to energy transport. Specifically, I aimed to (i) assess interannual variation in energy fluxes associated with waterbird guilds, (ii) evaluate long-term temporal trends in key energy-balance metrics, and (iii) examine whether changes in open water area are linked to variation in net energy balance. I hypothesised that waterbird guilds usually function as net energy-import or -export vectors in wetland ecosystems, but that the magnitude and direction of energy transport vary strongly among years in response to environmental and guild-structure dynamics.

## Methods

### Study site

**Fig. 1:**
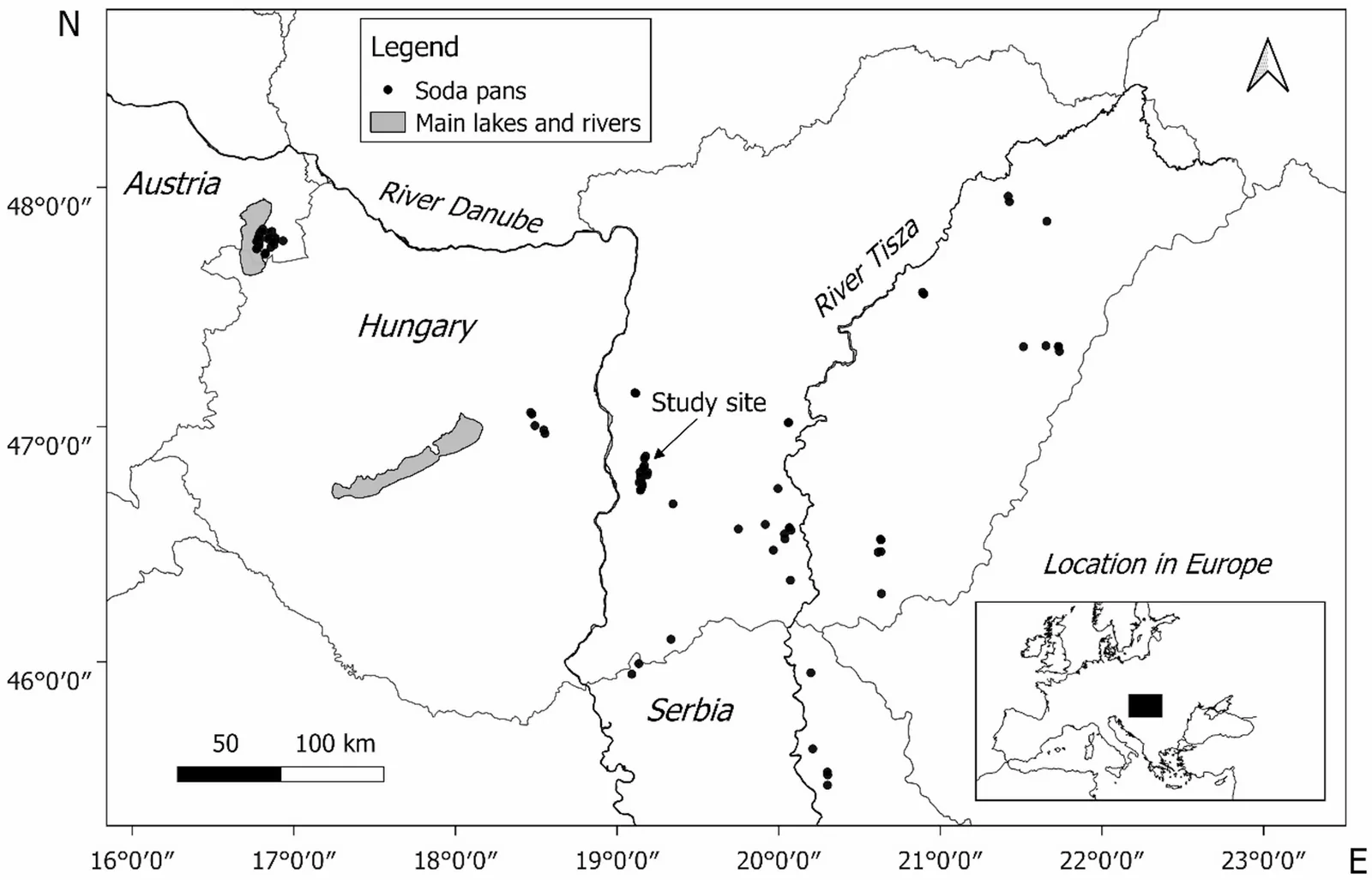
Location of the investigated soda pans in the Carpathian Basin. Data source of soda pan points is Boros et al.,^26^. The map was generated using QGIS software, version 3.22 (https://www.qgis.org).

The studied soda pans are very shallow (mean depth < 0.5 m), endorheic, polymictic open water bodies located in Central Europe (Hungary), in the interfluve area of the Danube and Tisza rivers (Fig. 1). Their continental climate, influenced by both oceanic and Mediterranean effects, together with their shallowness, results in strong interannual and seasonal fluctuations in water level and temperature. The pans occur in closed basins where evaporation exceeds outflow, leading to intermittent hydroperiods and high concentrations of organic and inorganic compounds^25, 26^. They represent characteristic soda and soda-saline inland waters of Eurasia, dominated by sodium (Na^+^) and carbonate ions (HCO_3_^−^ + CO_3_^2−^), and occasionally chloride, in both ground- and surface waters^27^.

Two soda pans were selected based on long-term (1986–2017) waterbird census data: Kelemen-szék (46°47′N, 19°11′E), with open water cover ranging from 1 to 459 ha, and Zab-szék (46° 50′ N, 19° 10′ E), with open water cover between 1 and 334 ha. Both sites lie in the Carpathian Basin (Fig. 1) at the intersection of the East Atlantic and Black Sea–Mediterranean flyways, forming important non-breeding and stopover habitats for African–West Eurasian migratory waterbirds^28^. Accordingly, both pans are designated as Ramsar sites and Natura 2000 Special Protection Areas due to their internationally significant waterbird populations and as unique ecosystem globally.

These simplified unique soda pans serve as ideal natural laboratories for modelling avian cross-ecosystem energy vectors. In these temporary, ephemeral systems, the dual-control function (bottom-up and top-down) of waterbirds^3^ is particularly intensified due to specific environmental constraints: the absence of fish results in shortened food chains, while shallow water levels lead to exceptionally high specific densities of waterbirds relative to surface area and volume. These concentrated biotic interactions establish waterbirds as flagship indicators within these continental ecosystems, facilitating a simplified direct assessment of energetic transport between aquatic and terrestrial environments.

### Classification of waterbird nutrient cycling guilds

The Boros’s^19^ classification method for quantifying waterbird nutrient cycling guilds was applied as a methodological extension to energy-flow calculations in inland waters. This framework integrates feeding habitat, daily habitat use patterns and the quantitative role of waterbirds in nutrient and energy fluxes. I developed this classification based on extensive regional reference data on habitat selection, feeding behaviour, roosting and aggregation patterns^29, 9, 30, 21, 31^. It represents an improved and extended adaptation of the single nutrient-transport guild concept proposed by Oláh et al.,^21^.

Taxa (species or higher level of taxa) were classified based on feeding habitat (aquatic, wetland, terrestrial), trophic role and daily habitat-use patterns, summarised in Table 1. Accordingly, three functional guilds were defined: net importer (NIM), comprising species feeding mostly outside inland waters; importer–exporter (IMEX), comprising species feeding both inside and outside inland waters; and net exporter (NEX), comprising species feeding predominantly within inland waters^19^.

**Table 1:** The complex classification factors of the waterbirds used for quantification of specified nutrient cycling guilds^19^.

Nutrient-cycling guilds	Waterbird groups or species	Scientific name	Feeding habitats	Feeding guilds	Defacation time factor	Feeding time factor
NIM	Geese	*Anser*,* Branta spp.*	Terrestrial, wetland	Herbivorous	0.6	0
NIM	Eurasian Stone-curlew, Dotterel	*Burhinus oedicnemus*,* Charadrius morinellus*	Terrestrial, wetland	Invertebrates	0.6	0
NIM	Pratincoles	*Glareola spp.*	Terrestrial, wetland	Invertebrates	0.6	0
NIM	Cranes	*Grus*,* Leucogeranus spp.*	Terrestrial, wetland	Omnivorous	0.6	0
IMEX	Dabbling ducks	*Anas*,* Mareca*,* Spatula spp.*	Aquatic, wetland, terrestrial	Omnivorous	0.6	0.2
IMEX	Herons	*Ardea spp.*,* Botaurus stellaris*,* Bubulcus ibis*	Aquatic, wetland, terrestrial	Carnivorous	0.6	0.1
IMEX	Ruff	*Calidris pugnax*	Aquatic, wetland, terrestrial	Invertebrates	0.6	0.3
IMEX	Gulls	*Chroicocephalus*,* Hydrocoloeus*,* Ichthyaetus*,* Larus*,* spp.*	Aquatic, wetland, terrestrial	Omnivorous	0.6	0.1
IMEX	Storks	*Ciconia spp.*	Aquatic, wetland, terrestrial	Carnivorous	0.6	0.1
IMEX	Curlews	*Numenius spp.*	Aquatic, wetland, terrestrial	Invertebrates	0.6	0.1
IMEX	Glossy Ibis	*Plegadis falcinellus*	Aquatic, wetland	Carnivorous	0.6	0.1
IMEX	Big Plovers, Lapwings	*Pluvialis spp.*,* Vanellus vanellus*	Aquatic, wetland, terrestrial	Invertebrates	0.6	0.2
NEX	Small sandpipers	*Actitis*,* Calidris spp.*	Aquatic, wetland	Invertebrates	1	0.6
NEX	Small Herons	*Ardeola*,* Ixobrychus*,* Nycticorax spp.*,* Egretta alba*	Aquatic, wetland	Carnivorous	1	0.6
NEX	Diving ducks	*Aythya*,* Bucephala*,* Clangula*,* Melanitta*,* Mergus*,* Mergellus*,* Somateria spp.*	Aquatic	Omnivorous	1	0.6
NEX	Small Plovers	*Charadrius spp.*	Aquatic, wetland	Invertebrates	1	0.6
NEX	Skuas, Terns	*Chlidonias*,* Hydroprogne*,* Stercorarius*,* Sterna spp.*	Aquatic, wetland, terrestrial	Carnivorous	1	0.6
NEX	Swans	*Cygnus spp.*	Aquatic, wetland	Omnivorous	1	0.6
NEX	Coots, Crakes, Moorhen, Rails	*Fulica*,* Gallinula*,* Porzana*,* Rallus*,* Zaporina spp.*	Aquatic, wetland	Omnivorous	1	0.6
NEX	Snipes	*Gallinago*,* Lymnocryptes spp.*	Aquatic, wetland	Invertebrates	1	0.6
NEX	Black-winged Stilt, Pied Avocet	*Himantopus himantopus*,* Recurvirostra avosetta*	Aquatic, wetland	Invertebrates	1	0.6
NEX	Godwits, Dowitchers	*Limosa*,* Limnodromus spp.*	Aquatic, wetland	Invertebrates	1	0.6
NEX	Cormorants	*Phalacrocorax*,* Microcarbo spp.*	Aquatic	Piscivorous	1	0.6
NEX	Phalarops	*Phalaropus spp.*	Aquatic, wetland	Invertebrates	1	0.6
NEX	Eurasian Spoonbill	*Platalea leucorodia*	Aquatic	Carnivorous	1	0.6
NEX	Grey Plover, Eurasian Oystercatcher, Ruddy Turnstone	*Pluvialis squatarola*,* Haematopus ostralegus*,* Arenaria interpres*	Aquatic, wetland	Invertebrates	1	0.6
NEX	Grebes, Loons	*Podiceps*,* Tachybaptus*,* Gavia spp. spp.*	Aquatic	Piscivorous	1	0.6
NEX	Shelducks	*Tadorna spp.*	Aquatic, wetland	Omnivorous	1	0.6
NEX	Big sandpipers	*Tringa*,* Xenus spp.*	Aquatic, wetland	Invertebrates	1	0.6

Groups and species names after Gill et al.,^32^ (defacation time factor: species-specific defecation occurring on water: 0−1; Feeding time factor: species-specific feeding occurring on water.

Following this framework, total net energy import mediated by waterbird guilds was calculated as the sum of energy imported by NIM guilds and the import component of IMEX guilds (1):

1 $$\mathit{Net\ import}_{\mathrm{(waterbirds)}} = \mathrm{NIM}_{\mathrm{(import)}} + \mathrm{IMEX}_{\mathrm{(import)}}$$

Total net energy export was calculated as the sum of energy exported by NEX guilds and the export component of IMEX guilds (2):

2 $$\:{Net\ export}_{\:\left(waterbirds\right)}=\:{NEX}_{\:\left(export\right)}+\:{IMEX}_{\:\left(export\right)}$$

Total energy transport mediated by waterbirds was defined as the difference between net import and net export (3):

3 $$\:{\begin{array}{c}Total\ energy\:\\\:transport\end{array}}_{\:\left(waterbirds\right)}=\:{\begin{array}{c}Net\ energy\\\:import\end{array}}_{\:\left(waterbirds\right)}-\:\:\:\:{\begin{array}{c}Net\ energy\\\:export\end{array}}_{\:\left(waterbirds\right)}$$

Positive values indicate net energy import, while negative values indicate net energy export.

### Estimating net energy import of waterbirds

Daily excreted carbon (fecal + urate carbon) was estimated at species level using body-mass-based allometric relationships. Basal metabolic rate (BMR) was calculated following avian allometry^33, 34^ and converted to daily energy units (4):

4 $$\:{BMR}_{\left(kJ/day\right)}=\:3.79\cdot \:{M}_{\left(kg\right)}^{0.723}\cdot 86.4$$

Body mass values were taken from the AVONET database at the species level^35^. Daily energy expenditure (DEE) was estimated as (5), using an activity multiplier f ranging from 2.0 to 3.5^36, 37^:

5 $$\:DEE=f \cdot BMR$$

Energy was converted to carbon assuming 1 g C ≈ 39.8 kJ^38, 39^. Fecal carbon was estimated using guild-specific assimilation efficiencies^40^ (6):

6 $$\:{C}_{(feces)}=\frac{DEE\:}{\:39.8}\cdot \frac{1-AE}{AE}$$

Urate carbon was calculated assuming protein-based metabolism and uric acid excretion^41, 42^. Total daily excreted carbon was calculated as (7):

7 $$\:{C}_{\:\left(excreted\right)}=\:{C}_{\:\left(feces\right)}+\:{C}_{\:\left(urate\right)}$$

Waterbirds were counted monthly during daylight, except during dry or frozen periods. Monthly counts were treated as average daily densities. Net daily energy import was estimated from NIM and IMEX guilds using (8):

8 $$\:{Net\ import\:}_{(kJ/ha/day)}=\sum\:_{(guild\:NIM+IMEX)}\:[\:{D}_{\:\left(ind/ha/day\right)}\:\cdot {C\:}_{\left(g/n/day\right)}\cdot 39\:\cdot \:{T}_{\:\left(01\right)}]$$

where D = cumulative average daily density of waterbirds in guilds NIM and IMEX (individuals / hectare); C = daily carbon intake per individual (g C / day / bird number); 39 = energy conversion factor (kJ / g C); T = species-specific time factor of defecation occurring on water (0−1).

The species-specific time factor (T) represents the proportion of a 24-hour daily cycle that an individual spends on the water surface. It is derived as the ratio of the cumulative time spent on the water to the total duration of the day (24 h). These residency values were determined based on local field observations and the habitat-use patterns of GPS-tagged individuals, as summarized in Table 1.

### Estimating net energy export of waterbirds

Energy export was estimated from the energetic demand of waterbirds feeding within the aquatic system. Field metabolic rate (FMR) was calculated following Nagy^37^ (9):

9 $$\:{FMR}_{\left(kJ/day\right)}=\:10.5\:\cdot {BM\:}_{\left(g\right)}^{0.681}$$

Net energy export was calculated as (10) based on Boros’s guild quantification method^19^:

10 $$\:{Net\ export\:}_{(kJ/ha/day)}=\sum\:_{(guild\:IMEX+NEX)}\:[\:{D}_{\:\left(ind/ha/day\right)}\cdot {FMR}_{\left(Kj/day\right)}\cdot \:{T}_{\:\left(01\right)}]$$

where D = cumulative average daily density of waterbirds in guilds IMEX and NEX (individuals / hectare); FMR = field metabolic rate (kJ/day); T = species-specific time factor of feeding occurring on water (0−1); BM = body mass of bird species (g).

### Percent energy balance

Percent Energy Balance (%) of waterbirds (PEB _waterbirds_) was calculated as (11) based on guild transport index method used^43^:

11 $$\mathrm{PEB}_{\mathrm{(waterbirds)}} = \left( \frac{\text{Net import} - \text{Net export}}{\text{Net import} + \text{Net export}} \right) \ \cdot 100$$

Positive values indicate net energy import, negative values net energy export.

The calculations, statistical analyses and figures were prepared with the R program package.

### Statistical analyses

Statistical analyses and figure editing were performed in the R environment. The normality of the energetic parameters (net import, net export, and energy balance) was assessed using Shapiro-Wilk tests. As the data distributions significantly deviated from normality (*p* < 0.05), non-parametric procedures were applied. Differences between energy input (import) and output (export) were analyzed using paired Wilcoxon signed-rank tests. To determine if the PEB_waterbirds_ (%) deviated significantly from zero, a one-sample Wilcoxon signed-rank test was employed. Statistical significance was set at *p* < 0.05.

Temporal trends were analysed using generalized additive models (GAMs) in R (mgcv package) based on Wood’s method^44^. Models with common or site-specific temporal smooths were compared using Akaike’s Information Criterion. Overall change was quantified as the difference between fitted values in the first and last year. Mann–Kendall tests and Sen’s slope estimates were used as complementary monotonic trend analyses. Spearman rank correlations were calculated using asymptotic p-values.

## Results

### Composition of the waterbird community

**Fig. 2:**
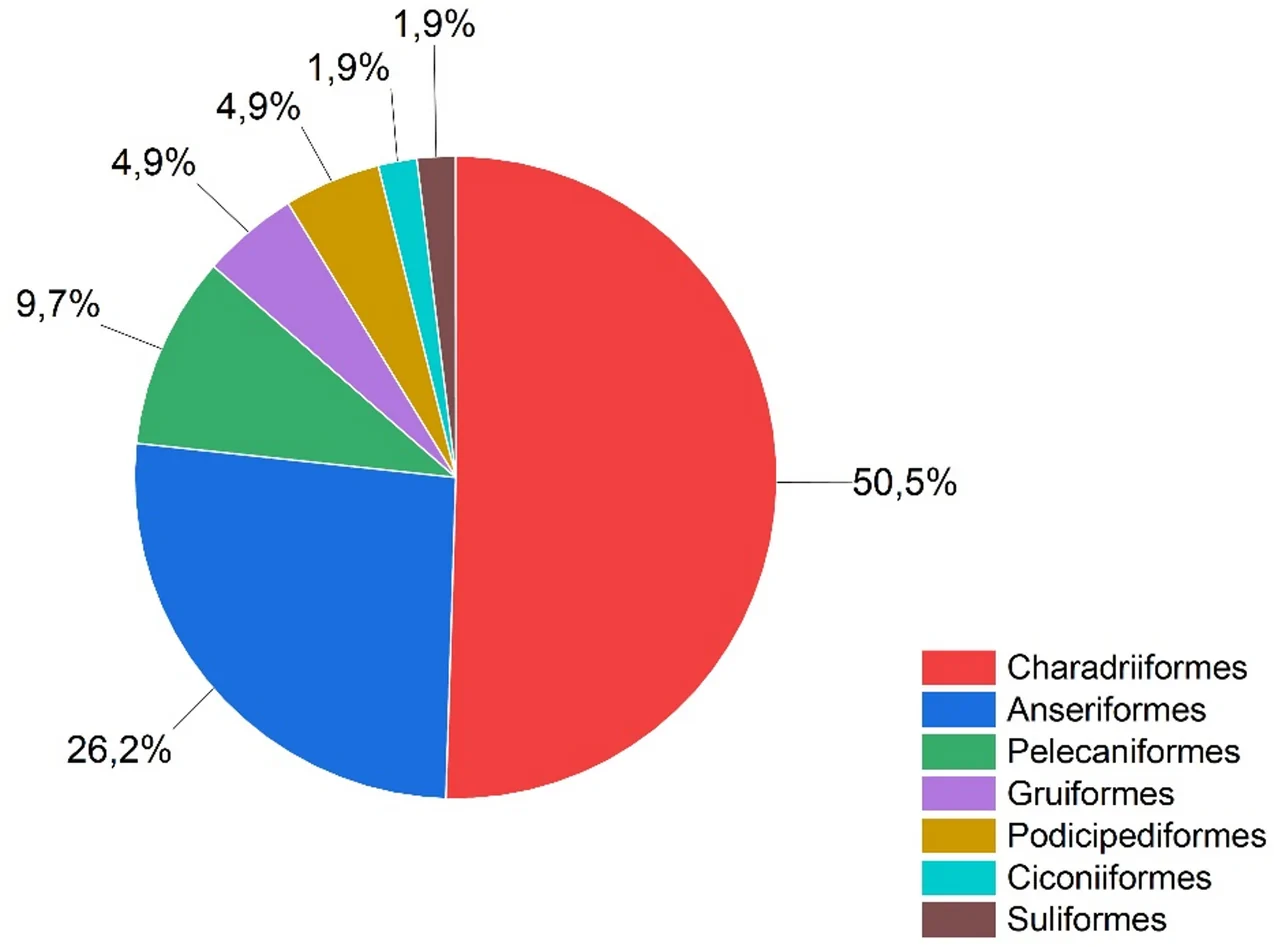
Taxonomic (Order) composition of the waterbird community based on the number of species in the studied soda pans.

The waterbird community recorded in the studied soda-saline wetlands comprised a total of 103 unique species. Regarding taxonomic distribution based on the number of species, the community was predominantly characterized by two major orders: Charadriiformes (shorebirds, gulls, and terns), representing nearly half of the species richness (50,5%; 52), followed by Anseriformes (waterfowl) (26.2%; 27): The other orders contributed smaller proportions to the overall avian diversity, such as Pelecaniformes (9.7%; 10), Gruiformes (4.9%; 5), Podicipediformes (4.9%; 5), while Ciconiiformes (1.9%; 2) and Suliformes (1.9%; 2). The taxonomic (Order) composition of the waterbird community is presented in Fig. 2 based on the number of species in the studied soda pans.

Furthermore, nutrient cycle guild classification revealed a clear dominance of the net exporter guild (primarily invertivores and piscivores), which encompassed 68.0% of the species, while the importer-exporter guild (omnivores and terrestrial foragers) and the net importer guild (primarily herbivores) represented 20.4% and 11.7% of the recorded community. Considering the entire study area between 1986 and 2017, the overall mean waterbird density was 117.88 ± 159.62 ind/ha (mean ± SD), with a median of 57.96 ind/ha and a range of 1.00 to 1378.50 ind/ha. A comprehensive summary of the waterbird community, including species-specific taxonomic data, nutrient cycle guilds, and total observed counts, is provided in Appendix 1.

### Net energy import, export and balance

Estimated annual specific net energy import and export (kJ/ha/yr), as well as their balance, for the two investigated soda pans during the 1986–2017 period are summarised in Table 2. Over the twenty analysed years spanning three decades, both waterbird-mediated energy import and export varied across several orders of magnitude. Net energy import ranged widely from 574,712 to 57,022,011 kJ/ha/yr, while net energy export from 361,206 to 16,171,538 kJ/ha/yr.

**Table 2:** The results of the estimated specific yearly net energy import, export (kJ/ha/year) and Percent Energy Balance (%) of waterbirds (PEB_waterbirds_) in twenty-year (N_year_=20) examined over a three-decade period (1986−2017) period on the two investigated soda pans.

Sites	Years	Mean of open water (ha)	Net import (kJ/ha/year)	Net export (kJ/ha/year)	PEB_waterbirds_ (%)
Kelemen-szék pan	1986	66	1 796 442	1 898 072	− 3
	1987	69	10 961 399	1 717 886	73
	1988	55	8 493 779	2 737 459	51
	1989	36	5 473 081	2 719 567	34
	1990	55	43 821 140	7 919 778	69
	1991	61	2 343 016	1 668 370	17
	1992	74	17 185 529	2 673 847	73
	1993	45	19 578 649	5 456 641	56
	1994	65	22 147 585	6 970 402	52
	1995	51	9 614 755	5 312 065	29
	1996	61	25 952 257	7 208 730	57
	1997	69	16 941 726	2 925 666	71
	1998	24	27 884 735	4 698 343	71
	1999	21	8 003 044	4 896 923	24
	2000	84	3 851 887	1 886 787	34
	2001	57	12 595 905	3 808 873	54
	2002	44	18 429 042	3 943 459	65
	2004	83	11 191 290	1 543 619	76
	2014	160	2 783 855	2 359 736	8
	2017	34	54 459 174	7 118 474	77
Zab-szék pan	1986	27	790 289	986 754	− 11
	1987	13	574 712	868 978	− 20
	1988	55	11 161 777	4 673 496	41
	1989	15	9 014 205	1 058 294	79
	1990	9	7 822 622	361 206	91
	1991	22	2 296 474	1 366 191	25
	1992	15	6 243 864	1 065 676	71
	1993	18	2 304 068	559 590	61
	1994	23	20 013 279	4 901 708	61
	1995	8	13 014 033	16 171 538	− 11
	1996	13	16 845 222	2 308 799	76
	1997	24	15 611 190	3 046 436	67
	1998	16	4 563 950	3 112 340	19
	1999	16	34 236 644	10 097 025	54
	2000	55	21 863 489	4 648 080	65
	2001	36	57 022 011	3 237 791	89
	2002	44	16 728 187	2 726 271	72
	2004	70	14 092 930	1 200 184	84
	2014	144	9 587 456	1 170 647	78
	2017	58	12 267 099	1 944 958	73
	Mean	47	15 327 317	3 668 528	51
	Min.	8	574 712	361 206	− 20
	Max.	160	57 022 011	16 171 538	91

**Fig. 3:**
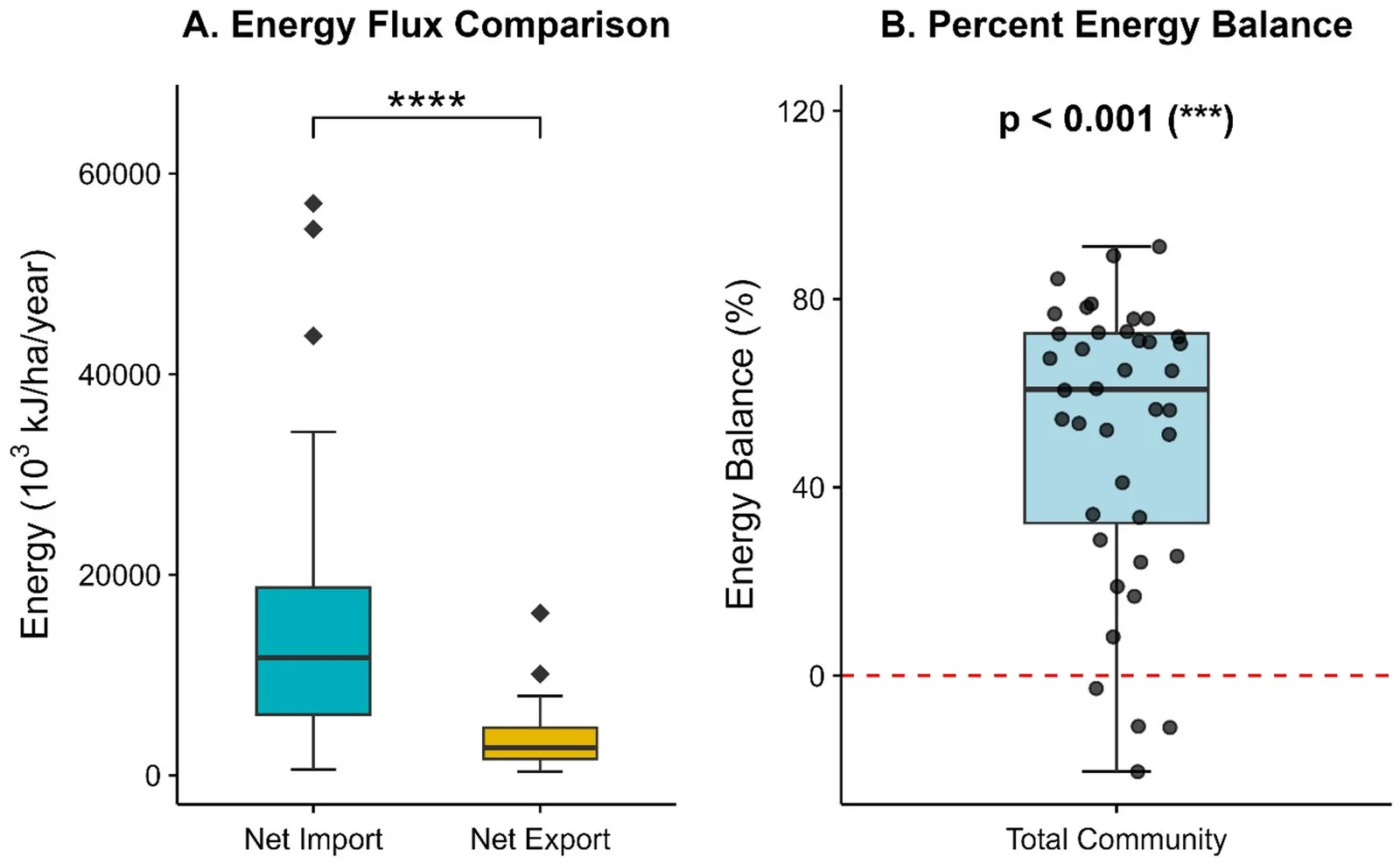
Energy flux and percent energy balance of the waterbird community. (**A**) Comparison of energy flux by net energy import and export (kJ/ha/year). (**B**) Percent Energy Balance (%) of waterbirds (PEB_waterbirds_). Boxplot components: center line = median; box = interquartile range (IQR, 25–75%); whiskers = 1.5 × IQR range; diamonds (♦) = statistical outliers; points (•) = individual years/sites.

The PEB), used here as an index of avian cross-ecosystem energy transport, varied between − 20% and 91%, with a mean value of 51%. Positive PEB values indicate years in which waterbird assemblages functioned predominantly as net energy-import vectors at the ecosystem scale. Accordingly, a highly significant difference was observed between the amount of energy imported and exported by birds during the study period (Wilcoxon test, *p* < 0.001). Net energy import consistently and significantly exceeded net energy export. Consequently, the average community-level energy balance was significantly positive (+ 51.3%, *p* < 0.001), confirming that the studied soda pans function as net energy sinks regarding waterbird-mediated nutrient transport (Fig. 3).

### Temporal trends

Trend analyses revealed predominantly weak or non-significant long-term temporal patterns across most energy-balance variables. Model selection based on AIC indicated that some variables were better described by site-specific temporal smooths, whereas others followed a common temporal trajectory across sites. For the majority of variables, estimated smooth terms were not statistically significant, indicating that interannual variability outweighed sustained directional change.

In contrast, significant temporal effects were detected for mean open water area (ha) and net energy import per unit area (kJ/ha/yr). For these variables, GAM smooth terms indicated consistent temporal structure, suggesting systematic change over the study period. GAM-based overall change metrics supported these results, revealing clear net directional shifts between the first and last years of the time series. Non-parametric Mann–Kendall tests were broadly consistent with these findings, although the direction of monotonic trends could not always be robustly estimated at both sites.

**Fig. 4:**
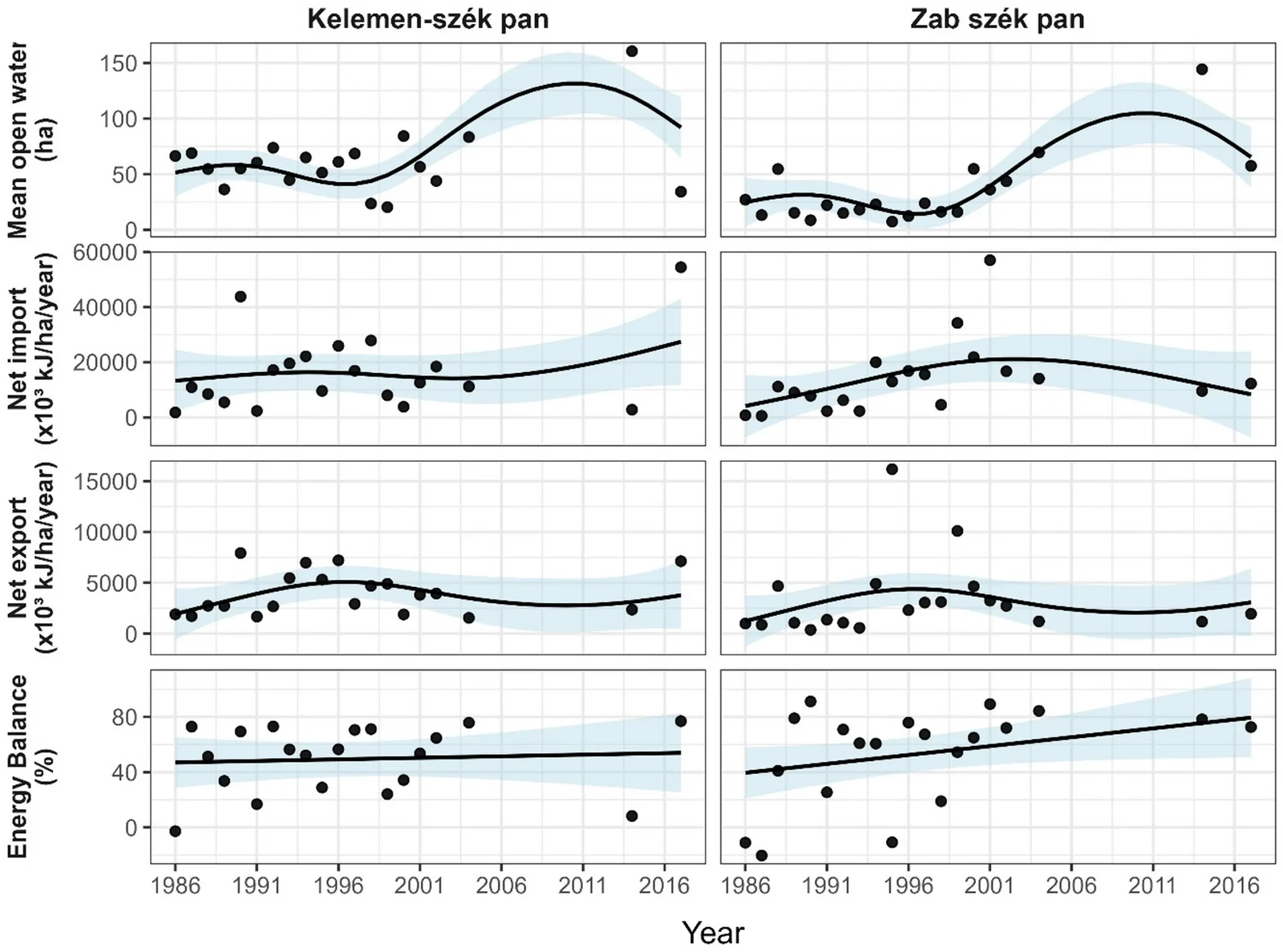
Temporal trends in energy import, export and Percent Energy Balance (%) of waterbirds (PEB_waterbirds_) at the two investigated sites (*N* = 40) between 1986−2017.

Overall, the results indicate that while most energy-balance metrics remained temporally stable, selected variables exhibited significant temporal dynamics, highlighting variable-specific sensitivity to long-term environmental change (Fig. 4). Mean open water area was not significantly correlated with net energy import, net energy export or PEB when data from both sites were pooled (Spearman’s ρ = 0.07, − 0.01 and 0.08, respectively; all *p* > 0.6).

## Discussion

Quantifying how migratory animal guilds transfer energy across ecosystem boundaries remains a major challenge in ecosystem ecology. The results demonstrate that waterbird-mediated energy import and export in the investigated soda pans exhibited extremely high interannual variability, spanning several orders of magnitude. Such pronounced fluctuations indicate that the energetic role of waterbirds is strongly contingent on year-specific conditions, including hydrological regime, food availability, and the composition and density of waterbird assemblages. Importantly, this variability does not reflect random noise, but rather the inherently dynamic nature of waterbird energy-transport guilds, whose net energetic effects emerge from the balance between import and export processes operating simultaneously within and across species.

Despite this strong variability, the predominance of positive PEB_waterbirds_ values indicates that waterbird guilds frequently functioned as net energy-import vectors at the ecosystem scale, because the net energy import consistently and significantly exceeded net energy export. Consequently, the average community-level energy balance was significantly positive (+ 51.3%), confirming that the studied soda pans function as net energy sinks regarding waterbird-mediated nutrient transport. This suggests that, over multi-annual time frames, the studied soda pans often acted as net energy sinks mediated by avian movements, independently of short-term hydrological fluctuations. These findings are consistent with previous studies demonstrating that waterbirds can induce guanotrophication^45, 10^ and promote net heterotrophy in shallow, intermittent waters^29, 3, 46^. However, by explicitly quantifying both import and export components, my results extend this perspective by showing that waterbirds simultaneously exert bottom-up and top-down energetic influences, mediated by functionally distinct guilds. As waterbirds are flagship species and dual-control indicators of trophic structure in Eurasian soda-saline waters^3^, soda pans provide an ideal system for modelling avian cross-ecosystem energy transport. However, a notable discrepancy exists between the species richness of nutrient-cycling guilds and their actual energetic impact. The functional asymmetry between guilds ensures that energy import remains dominant over export regardless of species richness, fundamentally defining the ecosystem’s role as a significant energy sink.

Waterbirds can thus act both as energy importers and exporters within food webs, with these roles occurring concurrently through different species or guilds within the same ecosystem, or through the same species across different ecosystems. Many existing models of avian effects in aquatic systems have focused primarily on top-down control mechanisms, particularly predation by piscivorous birds^47, 48, 49^. Classic examples of net energy export include seabird colonies, where piscivorous birds transport large amounts of marine-derived energy onto land through guano deposition^14, 15, 16^. In contrast, the potential for waterbirds to act as net energy importers into inland waters has often been underestimated relative to internal aquatic production^50, 4, 6^. The results demonstrate that both energy import and export can be substantial in inland wetlands, and that their net balance depends on guild composition, behaviour and habitat use rather than on a single trophic mechanism.

The absence of strong or consistent long-term temporal trends in most energy-balance variables further indicates that short-term variability outweighs gradual directional change, a pattern commonly reported in highly dynamic shallow lake and wetland systems, particularly in intermittent soda pans^26, 51^. Consequently, I partly reject the hypothesis that net energy import responds predictably to long-term trends in open water area, but partly confirm as it is rather depends on guild-structure dynamics. Similar patterns have been observed in other wetland ecosystems, where biotic fluxes driven by migratory animals are characterised by episodic dominance and high temporal stochasticity rather than steady monotonic change^52, 8^.

Notably, mean open water area was not significantly correlated with net energy import, net energy export or PEB, indicating that habitat extent alone is a poor predictor of waterbird-mediated energy fluxes. This finding is consistent with previous studies showing that energy transfer in aquatic ecosystems is primarily regulated by trophic interactions, prey accessibility and species-specific foraging behaviour, rather than by habitat size per se^53, 2^. Consequently, years with similar water surface areas may differ substantially in their energetic balance depending on the functional composition and behavioural dynamics of waterbird assemblages.

Together, these results highlight the complexity and context dependence of waterbird-driven energy dynamics in inland wetlands. Rather than acting solely as consumers within local food webs, waterbirds function as dynamic energy-transport guilds that transfer energy directionally across ecosystem boundaries. This perspective integrates avian movement ecology with ecosystem energetics and provides a mechanistic basis for understanding how migratory animals regulate cross-ecosystem energy flow in variable environments.

### Study limitations and uncertainty factors

The present study identifies two primary areas of uncertainty regarding the energetic estimates: Dietary Variability and Metabolic Proxies. Due to the high diversity of potential food sources, explicit dietary composition was not incorporated into the model. Instead, I utilized body mass-based metabolic rates—specifically BMR for energy input and FMR for energy export—as robust proxies for energy flux. While variations in food type can influence metabolic efficiency, the necessity for birds to maintain physiological energy homeostasis suggests that these allometric scaling models provide a balanced and reliable estimate of the overall magnitude of transport. Due to the high intraspecific and interspecific variability of avian diets, incorporating the explicit dietary composition of individual species directly into such a generalized model was not feasible. To address this limitation, dietary habits were accounted for indirectly through the classification of feeding guilds, which served as the basis for defining nutrient-cycling guilds. Species were categorized into five primary feeding guilds: herbivores, omnivores, carnivores, piscivores, and invertebrate-eaters (detailed in Table 1). These nutrient-cycling guilds were subsequently integrated into both the energy import and export calculations. Consequently, while precise dietary choices were not modeled at the species level, the functional role of diet was indirectly incorporated in a guild-specific manner.

Age or sex specific factors: My calculations rely on taxon-specific data without accounting for age- or sex-structured differences within populations. While the omission of age- or sex-specific metabolic requirements is a recognized limitation, its impact is expected to be negligible at the scale of the entire waterbird community. At this higher level of organization, individual demographic variations likely balance out, and the resulting error remains minor relative to the total community-level energy transport.

## Conclusions

This study reveals that waterbirds act as dynamic cross-ecosystem energy-transport guilds, playing a more central role in ecosystem energetics than previously recognised, especially in migrating hotspots e.g. Ramsar-sites. By jointly quantifying energy import, export and net balance at the community level, I show that waterbird-mediated energy fluxes are highly variable through time, yet most often result in net energy import into inland wetlands.

Crucially, the direction and magnitude of these fluxes are determined by the functional composition, behaviour and habitat use of waterbird assemblages rather than by simple physical attributes such as open water area. Energy import and export usually operate simultaneously within the same ecosystem, mediated by different guilds or by the same species under contrasting environmental conditions.

By extending nutrient-cycling guild concepts into the energy flow over ecosystems, the proposed framework provides a scalable approach for quantifying animal-mediated energetic redistribution across ecosystems. These findings challenge the prevailing view of waterbirds as solely local consumers or nutrient vectors and instead position migratory animals as directional energy vectors that couple aquatic and terrestrial ecosystems. Incorporating energy-transport guilds into ecosystem energetics creates new opportunities to link movement ecology, food-web dynamics and spatial subsidy theory, particularly in highly variable environments where physical habitat metrics alone fail to predict ecosystem functioning.

## Supplementary Information

Below is the link to the electronic supplementary material.

Supplementary Material 1

## Data Availability

The datasets generated and analyzed during the current study are available from the corresponding author on reasonable request.
